# Open Healing: A Minimally Invasive Protocol with Flapless Ridge Preservation in Implant Patients

**DOI:** 10.3390/biology11010142

**Published:** 2022-01-14

**Authors:** Alecsandru Ionescu, Aliona Dodi, Lucian Cristian Petcu, Mihnea Ioan Nicolescu

**Affiliations:** 1 Aesthetics One Dental Center, 021387 Bucharest, Romania; alecs.ionescu@aestheticsone.ro (A.I.); aliona.dodi@aestheticsone.ro (A.D.); 2 Faculty of Medicine, Ovidius University of Constanta, 900527 Constanta, Romania; petculucian@univ-ovidius.ro; 3 Division of Histology, Faculty of Dental Medicine, “Carol Davila” University of Medicine and Pharmacy, 050474 Bucharest, Romania; 4 Laboratory of Radiobiology, “Victor Babeș” National Institute of Pathology, 050096 Bucharest, Romania

**Keywords:** guided tissue regeneration, bone regeneration, tissue level dental implants, flapless, wound healing, CBCT, ridge preservation

## Abstract

**Simple Summary:**

We describe a minimally invasive technique for optimal preservation of post-extraction tooth socket, prior to implant insertion in over 100 patients (*n* = 104, with 0.55 sex ratio), with a follow-up period of up to five years. This “open healing” concept is a flapless protocol, using a collagen membrane and a bovine bone substitute that yielded an uneventful healing, with sufficient bone formation, checked periodically after one, two, and five years by calibrated computer tomography. Open-healing protocol led to alveolar ridge and height preservation that contributed to a 98.5% implant survival and 94.8% success rate at five-year follow-up.

**Abstract:**

We aimed to validate the safety and efficacy of the minimally invasive “open healing” flapless technique for post-extraction socket and alveolar ridge preservation, while assessing the alveolar bone changes. The study enrolled (*n* = 104) patients (0.55 sex ratio), with atraumatic extraction of (**N = 135**) hopeless teeth, followed by either immediate placement of tissue level implants (**N_1_ = 26**), or later stage implant insertion (**N_2_ = 109**). No flap was raised in either situation. Post-extraction sockets were filled with deproteinized bovine bone granules and covered by collagen resorbable membrane—left purposely exposed during healing. This yielded an uneventful healing, with sufficient bone formation, while avoiding soft-tissue problems. The need for additional augmentation was assessed clinically and by calibrated CBCT scans at six months, before either loading (**N_1_**) or implant insertion (**N_2_**). Implant success and survival rate were evaluated at 12-, 24-, and 60-month follow-up control sessions. The inserted implants had a survival rate of 98.5% and a success rate of 94.8% at five-year follow-up. Open healing technique with flapless approach can be favorable for preserving the 3D architecture of the post-extraction socket, as well as the alveolar ridge width and height.

## 1. Introduction

Functional and aesthetic rehabilitation in implant-prosthetic therapy has become one of the main objectives in modern dentistry, and the demand for fast and minimally invasive implant procedures is continuously growing. The development of clinical protocols for early or immediate implant placement and loading can provide patients with the desired short treatment durations [1]. However, the first steps in the transition from a hopeless tooth to an implant supported restoration are tooth extraction and management of the post-extraction alveolae. Knowing the damage that ensues and the potential complications of tooth extraction, modern dentistry is moving toward socket protection and regeneration [2]. Although different studies have showed that immediate implantation will not prevent resorption of the alveolar ridge, as previously thought [3], the use of minimally invasive techniques without raising muco-periosteal flaps, combined with augmentation procedures in the gap formed between the implant and the alveolar walls, have led to ridge preservation, osseointegration, and long-term stability of peri-implant hard and soft tissues [4].

Nowadays, guided bone regeneration (GBR) is the preferred technique among alveolar ridge augmentation procedures [5]. From the available bone substituents, deproteinized bovine bone mineral with 10% collagen (DBBM-C) has shown very good results over time in alveolar ridges preservation and regeneration. Histological analyses showed a low rate of resorption of DBBM-C, generating long-term volumetric stability of augmented sites [6]. DBBM-C is often used in conjunction with semipermeable barrier membranes. According to the guided tissue regeneration protocols, membranes are used to protect the graft and prevent epithelial cells from entering the bone defect, a situation in which the process of bone neoformation could be altered [7]. In general, it is recommended to place the membrane beneath the flap and obtain a complete primary wound closure, without any tension. However, in augmentation procedures, primary flap closure can be difficult, requiring periosteal incisions and excessive soft tissue mobilization, increasing morbidity, generating local and regional inflammation, and an increased rate of dehiscence due to low blood supply in the already thin flap. In addition, the reduction of the buccal corridor, the lack of keratinized tissue, or the presence of scars can compromise the aesthetic result, requiring additional soft tissue surgery. In this context, a possible approach that could avoid the mobilization of adjacent soft tissues is the stabilization of the membrane without covering it by the flap margins and obtaining a healing by secondary intention [8,9]. Taking into account previously reported results [9,10,11,12,13,14], we chose to place the collagen membrane above the biomaterial introduced into the bone defect, maintaining and stabilizing it by fixing with continuous suture at the free gingival margins, without rising a mucoperiosteal flap, thus generating a *per secundam* healing of the augmented alveolar ridge.

## 2. Materials and Methods

There were (*n* = 104) patients included in the study (0.55 sex ratio) between 2014 and 2020 in a private clinic. All patients signed an informed consent. The mean patient age was 46.57 ± 12.68 years (aged 25–81 years). All patients had good health conditions with either no associated medical problems (28.15%) or systemic diseases with a well-balanced status (71.85%). Patients included in the study were both non-smokers (80.74%) and smokers (19.26%). Patients that had quit smoking more than six months before enrolling the study were included in the non-smoking group. Exclusion criteria were the presence of a serious medical condition, severely immune depressed patients, as well as obvious contraindications for radiological exposure—i.e., pregnant women.

Initial surgery consisted of atraumatic extraction of (**N = 135**) hopeless teeth with implant-prosthetic restoration planning. We used TRI Octa^®^ tissue level implants (TRI Dental Implants Int. AG, Hünenberg, Switzerland) in all cases. Prophylactic antibiotics were prescribed according to the clinic’s protocol and patient’s medical history (Amoxicillin/Clavulanic Acid 875 mg/125 mg, one tablet, twice a day for five to seven days, or Clindamycin 300 mg, one tablet, three times a day, for five to seven days).

**Figure 1:**
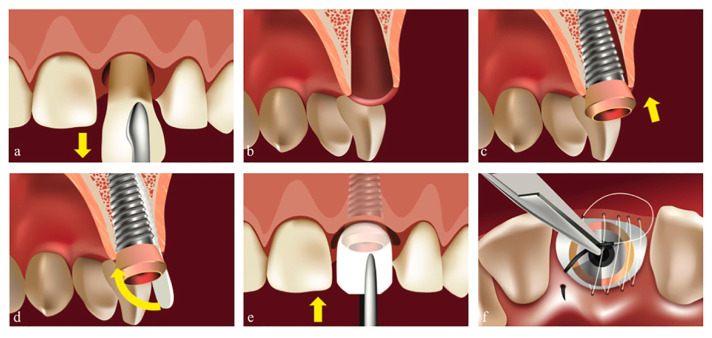
Immediate implant insertion (**N_1_ group**). Original illustration. (**a**) Atraumatic tooth extraction. (**b**) Post-extraction alveola. (**c**) Implant insertion in the same stage. (**d**) Shaped collagen membrane positioning between the implant and the delimiting walls, with bone granules filling the gap. (**e**) The membrane covering the graft and the implant. (**f**) PTFE continuous suture at free gingival margin level, stabilizing the membrane.

If the clinical situation allowed to obtain the primary stability with a predictable esthetic and functional outcome, the first option was implant placement in the same stage (Figure 1). Thus, in (**N_1_ = 26**) instances, immediately after the atraumatic extraction, we performed socket preservation with DBBM: Geistlich Bio-Oss^®^ (Geistlich Pharma AG, Wolhusen, Switzerland) and native bilayer collagen membrane (NBCM): Geistlich Bio-Gide^®^ (Geistlich Pharma AG, Wolhusen, Switzerland) in conjunction with the insertion of tissue level implants and “open healing” protocol for closing the gap surrounding the implant. We used a continuous polytetrafluoroethylene (PTFE) suture at the free gingival margin (Coreflone^®^, Implacore Sp. Z.o.o., Poznan, Poland) to stabilize the collagen membrane.

**Figure 2:**
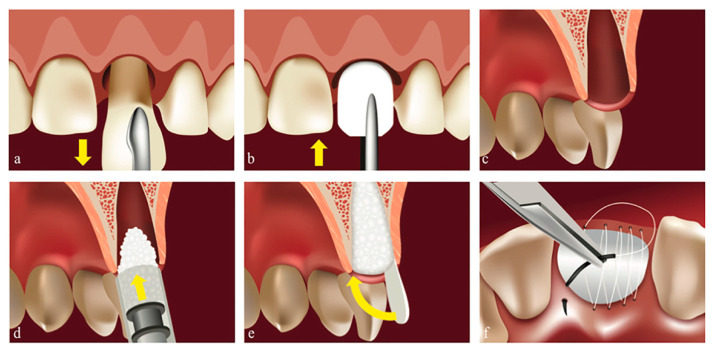
Post-extraction socket with all four delimiting walls (Situation **N_2_-1**). Original illustration. (**a**) Atraumatic tooth extraction. (**b**) Shaped collagen membrane positioning. (**c**) Alveola showing the integrity of the buccal wall. (**d**) Bone granules are highly condensed. (**e**) The membrane covering the graft. (**f**) PTFE continuous suture at free gingival margin level, stabilizing the membrane.

In the remaining (**N_2_ = 109**) cases, the implant was inserted in a later stage. In the **N_2_** group, the dental extraction was also performed with minimal trauma and with great care to preserve the buccal bone plate, without raising a flap, trying to keep the papillae and the surrounding soft tissue intact. After the extraction, the alveolae were carefully curetted. We inserted DBBM Geistlich Bio-Oss^®^ (Geistlich Pharma AG, Wolhusen, Switzerland) into the bone defect according to the manufacturer’s instructions, to provide a stable osteoconductive environment during healing. The granules were carefully compacted, so that an adequate space remained between them to allow the revascularization of the graft and the protein and growth factors intake necessary for the neo-osteogenesis process. For the consequent NBCM placement, we encountered two situations for the **N_2_** group: 

Situation **N_2_-1**: When the exposed site had all four delimiting lateral walls (Figure 2), we covered the biomaterial using NBCM Geistlich Bio-Gide^®^ (Geistlich Pharma AG, Wolhusen, Switzerland), stabilized by a continuous PTFE suture above, at the free gingival margins Coreflone^®^ (Implacore Sp. Z.o.o., Poznan, Poland). The main considerations for choosing these membranes were the long resorption time and high resistance to saliva.

**Figure 3:**
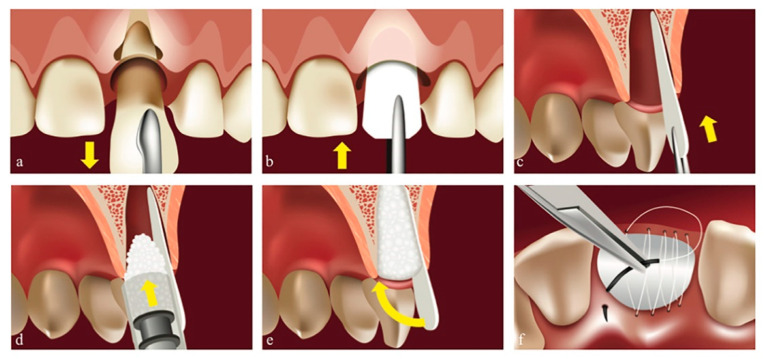
Post-extraction site with a missing lateral wall (Situation **N_2_-2**). Original illustration. (**a**) Atraumatic tooth extraction. (**b**) Shaped collagen membrane positioning. (**c**) Collagen membrane in situ. (**d**) Highly condensed bone granules are stabilizing the membrane apically. (**e**) The membrane covering the graft. (**f**) PTFE continuous suture at free gingival margin level, stabilizing the membrane.

Situation **N_2_-2**: When the exposed site had a missing lateral wall (most often the buccal one), we had to shape the membrane into a rounded angle trapezoid, placing the narrower part inside the alveola (Figure 3). There should be no deperiostation at this level, and the membrane should never be placed between the periosteum and the basal buccal bone area, to avoid compromising the vascular intake from the periosteum, which might lead to bone resorption. The narrow part of the membrane should be placed along the apical area of the alveola and stabilized between the granules filling the bone defect above and the basal bone below. The graft was moistened with blood from the alveola and thus the granules stabilized as well. The membrane should partially cover the proximal walls, and the wider part should remain outside the bone defect, cut towards the palate, and should be placed beneath the free gingival margin, thus completely covering the bone defect. We performed a PTFE continuous suture using Coreflone^®^ (Implacore Sp. Z.o.o., Poznan, Poland), stabilizing the collagen membrane with no tensions, favoring healing in conditions of mechanical peace.

The postoperative protocol included light rinsing with sage tea for the first 24–48 h, followed by light rinsing and/or washing with 0.2% chlorhexidine, supplemented with topical applications of chlorhexidine gel to the surrounding soft tissues, without applying the gel directly to the exposed membrane.

In all **N** cases (both **N_1_** and **N_2_** groups), sutures were removed after three weeks, thus allowing healing by secondary intention, and completely covering the bone defect with soft tissue. To allow the maturation of both soft and bone tissue, we waited for a healing period of four to six months before the next stage of treatment, which consisted either in implants loading (for **N_1_**) or implants placement, with or without additional surgical maneuvers (for **N_2_**).

Therefore, in this study we set evaluation timepoints for follow up in this study at specific intervals (Table 1). Protocol at the respective timeframes included clinical observations (evaluating inflammation, swelling, pain, and soft tissue healing) and cone beam computer tomograph (CBCT) scans and measurements (analyzing the bone volume/loss). 

**Figure 4:**
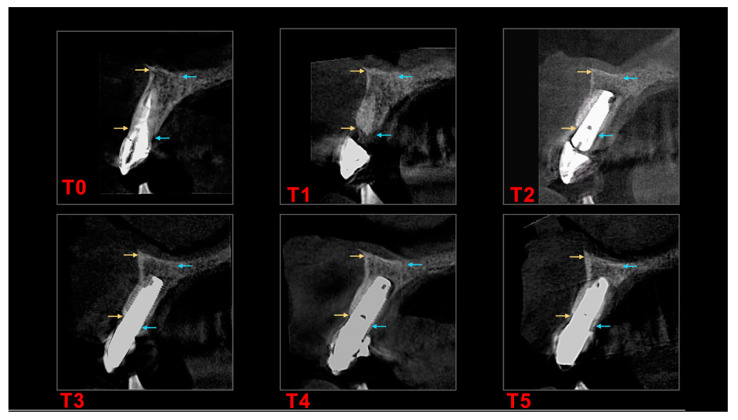
Example for CBCT measurement timepoints in **N_2_ group**. (**T_0_**) Initial situation. (**T_1_**) Before implant insertion. (**T_2_**) Just before implant loading. (**T_3_**) At 12 months after implant loading. (**T_4_**) At 24 months after implant loading. (**T_5_**) At 60 months after implant loading. Yellow arrows (buccal) and blue arrows (palatal) show reference points for calibrated measurements at the respective timepoints.

Since we only used tissue level implants, all implants were inside the 1.8–2.8 mm biological width. We assessed the resorption by CBCT measurements of bone wall height (vestibular–buccal “EXT” and oral–palatal/lingual “INT”) and alveolar ridge width (“Width”). We designated landmarks specific to each case, to reproduce the measurements in the control evaluations. For the immediate post-extraction implants, the second measurement was recorded before prosthetic loading. If, before the first surgical stage, one or more limiting walls were missing, the measurements were performed considering the existing bone level, and the treatment aimed to regenerate the affected tissues. Under these conditions, subsequent measurements used the same basal landmark, the crest landmark being given by the new bone level stabilized after healing. CBCT investigations (Figure 4) were done using a Cranex 3D^®^ tomograph (Soredex, KaVo Group, Tuusula, Finland), the parameters being standardized, considering international recommendations (6.3 mA; 90 kV; 200 micromillimeters Voxel). The measurements were performed and interpreted using dedicated software: OnDemand3D^®^ (Cybermed, Yuseong-gu, Daejeon, South Korea) by a single operator at timepoints specified in Table 1.

We allowed six months for healing time before loading the implants using fixed single unit and multiunit cemented prosthetic restorations, respecting the biological width protocol [15].

We respected the Misch implant success criteria [16], i.e., at least 12 month after loading—T_3_,T_4_,T_5_, and lack of pain and mobility. Furthermore, the survival and success rate of implants were analyzed for each timepoint [17], using as qualifying criteria the absence of: (1) persistent subjective complaints, foreign body sensations, dysesthesia; (2) infection and swelling around the implant; (3) mobility; and (4) continuous radiolucency around the implant. After these steps, the CBCT measurements were statistically analyzed.

For statistics we used IBM SPSS Statistics 23 and MedCalc 14.8.1 and the following procedures: descriptive statistics, graphs, nonparametric statistical tests (Chi-square test for association for two categorical variables, Chi-squared test for the comparison of two proportions) and parametric statistical tests (independent sample *t*-test, one-way ANOVA test). The significance level used for the tests was α = 0.05. A *p*-value less than α and a test statistic that falls within the critical region are the reason for rejecting the null hypothesis in favor of the alternative hypothesis.

## 3. Results

The implant losses were divided into early losses (one implant lost during the osseointegration period) and late losses (one other implant lost after the implants were osteointegrated and loaded), generating an overall survival rate of 98.52% after five years (i.e., 133 out of 135 implants). With respect to the previously mentioned criteria, we reported nine subjective complaints during the observation period, with no need for unplanned surgical treatment, generating a non-complaints rate of 93.33%. The subjective complaints were reported by sex (male 5.90%, female 0.70%), with proportions not differing significantly from each other at the 0.05 level for smoking habit, systemic diseases, or implant site positioning.

**Figure 5:**
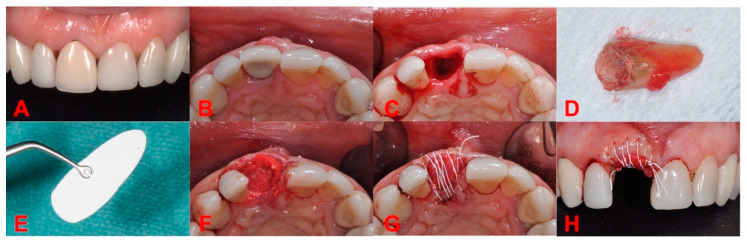
First surgery stage. (**A**) Initial situation (frontal view). (**B**) Initial situation (occlusal view). (**C**) Socket after atraumatic extraction. (**D**) Atraumatic extracted root. (**E**) Bio-Gide^®^ membrane shaped. (**F**) Bio-Gide^®^ membrane deeply inserted up to the basal bone, Bio-Oss^®^ granules are highly condensed after the membrane is placed. (**G**) Bio-Gide^®^ covering the socket open healing with PTFE continuous suture (occlusal view). (**H**) Bio-Gide^®^ covering the socket open healing with PTFE continuous suture (frontal view).

**Figure 6:**
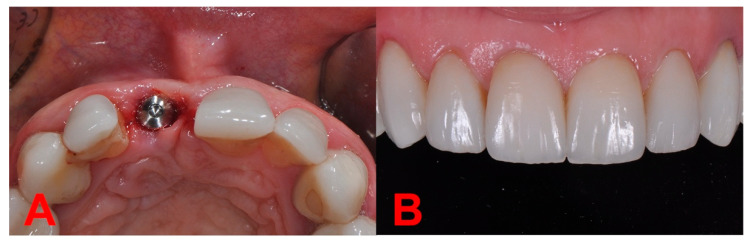
Flapless implant insertion after ridge healing. (**A**) Tissue level implant insertion. (**B**) Final restorations ceramic crowns and veneers.

During the observation period, a total of 104 patients (45.19% female and 54.81% male), with **N = 135** surgical sites were treated—85 (62.96%) in the maxilla, and 50 (37.04%) in the mandible (Table 2). During the first surgical stage, in almost a fifth of the cases implant was inserted immediately after the extraction—**N_1_ = 26** sites (19.26%), while the rest of **N_2_ = 109** sites (80.75%) received atraumatic extraction only (Figure 5) and implant placement in a later stage (Figure 6). In both situations, the flapless open healing protocol using DBBM and resorbable collagen membrane was performed, the main reason for immediate implant placement where primary stability could have been obtained being the predictability of the esthetic and functional outcome.

To demonstrate the safety and efficacy of the protocol, the first parameter analyzed was the occurrence of any complications during the healing period and the need for additional grafting, planned or not, following the use of this protocol. From the **N = 135** sites analyzed, although all of them showed uneventful early healing, only four sites (all from **N_2_**) needed additional unplanned surgery after the clinical and CBCT observations at six months (2.96%). All other 131 sites healed long-term as planned (97.04%). The patients who needed additional treatments were suffering of systemic diseases (Hepatitis, Type B/C). There was a functional reason for additional surgical maneuvers, namely the need of adequate ridge contour to facilitate implant placement in prosthetically driven position; as such, we performed a minimally invasive bone flapless splitting/spreading procedure to improve the buccal crest profile for a better mucosal integration of the future prosthetics. Nevertheless, all four sites—located posteriorly, two on the upper, and two on the lower jaw—received implants in this second stage.

By using the one-way ANOVA test, we confirmed (Table 3) that there are no significant differences between the buccal bone height mean values (mm) corresponding to the considered time moments T_0_–T_5_ (F = 1.124, *p* = 0.346 > α = 0.05). Levene’s test for equality of error variances shows equal group variances (Levene statistic = 1.750, df_1_ = 5, df_2_ = 750, *p* = 0.121).

There are also no significant differences between the oral bone height (palatal/lingual) mean values (mm)—Table 4, corresponding to T_0_–T_5_ (F = 0.529, *p* = 0.754 > α = 0.05), confirmed by one-way ANOVA test. In this case, Levene’s test showed equal group variances, too (Levene statistic = 1.390, df_1_ = 5, df_2_ = 750, *p* = 0.226).

However, we found significant differences between the alveolar ridge width mean values (mm)—Table 5, corresponding to T_0_–T_5_ (F = 4.375, *p* = 0.001 < α = 0.05) in the sense that the mean value at T_0_ differs statistically significantly from all other mean values obtained at T_1_–T_5_ (*p* < 0.05), while T_1_–T_5_ mean values showed no significant differences (*p* > 0.05) (Student–Newman–Keuls test for all pairwise comparisons). Moreover, Levene’s test showed equal group variances (Levene statistic = 0.421, df_1_ = 5, df_2_ = 750, *p* = 0.834). 

**Figure 7:**
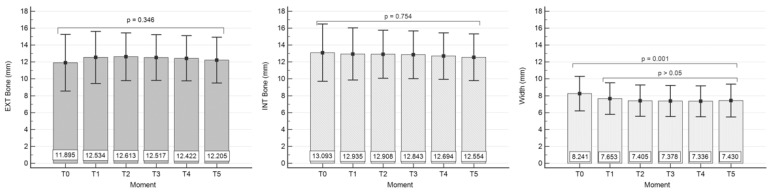
Graphical comparison of mean values of CBCT measurements. Left panel: buccal wall bone height (EXT Bone). Centre panel: oral (palatal/lingual) bone wall height (INT Bone). Right panel: Ridge width (Width). See Material and Methods section for T_0_–T_5_ time moments. All values are in mm.

For an easier statistics overview we have graphically summarized the above information in Figure 7.

## 4. Discussion

Open healing as dehiscence followed by open wound healing was considered for many years a complication of guided bone regeneration procedures, as stated in earlier studies, even though it did not significantly alter the final clinical outcome. Our study filled the gap of previous studies by reinforcing the idea that ridge augmentation using the open-healing concept as a well-designed technique is a predictable and minimally invasive regenerative procedure to create sufficient ridge volume suitable for prosthetically driven flapless implant placement with no additional soft tissue surgery needed and has the potential to become a general clinical option.

Our results showed that, for the **N_2_ = 109** sites where implants were inserted in a later stage, the “open healing” technique allowed the “flapless” implant placement without the need for further grafting. Previous results indicated the risk of a complication during the healing period after implant insertion is 4.42 times lower in the case of “flapless” insertion compared to a classical flap insertion [18].

Regarding the buccal bone height, our results showed no significant statistical difference of the variable “Ext Bone/mm” reported from T_0_ to T_1_ and from T_1_ to T_5_. At the same time, there are no statistically significant differences between the mean values of the variable “Int Bone/mm” representing the palatal/lingual bone wall height, corresponding to T_0_–T_5_. Instead, we found statistically significant differences between the average values of the variable “Width/mm” for the two groups, representing measurements at T_0_ and T_1_. However, no statistically significant differences were reported between the mean values of groups from T_1_–T_5_.

From a clinical point of view, these differences confirm that the bone remodeling is limited to a ridge width reduction after the atraumatic extraction, regardless of whether the implant was placed in the same stage or not, while the height of both buccal and palatal/lingual bone walls remained stable, confirming from a biological point of view the flapless approach.

We have considered that alveolar bone should be interpreted as a tooth-dependent structure, distinguishable from the rest of the jawbone [19]. Consequently, leaving as much of the teeth-related structures undisturbed leads to a higher rate of long-term success in maintaining a good proportion of the alveolar bone even after tooth extraction. Due to the biological advantages offered by the tissue level implants, after T_1_ (flapless insertion determination moment) and T_2_ (implant loading with respect to the biological width protocol), we noticed a ridge width stability associated with the other determination moments (T_3_–T_5_).

Given that all implants used were tissue level with the same prosthetic platform and considering that all other parameters were the same, we can conclude that the implant-prosthetic design concept is decisively contributing to the bone volume stability obtained with the described flapless surgical techniques. This conclusion completes the results of a recent study that analyzed the effect of different types of connections on the crestal bone, in order to obtain predictable and stable long-term results [20].

Furthermore, there were no associated statistical differences between the bone parameters evolution from T_0_–T_5_ and different variables like sex, smoking habits, systemic diseases, or the extracted teeth positioning as successional or accessional teeth.

In the attempt to reduce the need for advanced surgical procedures and to simplify the treatment plan, several surgical techniques were developed to reduce post-extractive alveolar atrophy. Socket/alveolar ridge preservation, with the application of different biomaterials, is the most common procedure aiming to control crestal bone resorption following dental extractions [21]. 

The minimally invasive technique that we described provides the necessary conditions for optimal healing in post-extraction sockets, regardless of the number of walls surrounding the bone defect, the evaluation of bone quality during treatment planning however being mandatory [22].

There are multiple protocols for ridge preservation, each specialist using them according to personal preferences, training, and experience. The existing studies cannot demonstrate the superiority of any biomaterial or technique [23]. It has long been considered that perfect flap closure and primary wound healing is necessary for successful bone graft integration [24]. In a study where primary closure was not achieved, the results showed partially preserved width and interproximal bone height of alveolar ridge [25], but the final outcome could have been influenced by grafting material and the multiple stage flapping protocol. However, other studies showed that there was no significant difference between flap or flapless interventions [26] in regard to the newly formed bone, residual graft, and bone trabeculae, suggesting that membrane exposure does not affect the regenerative process [27]. While full-thickness flaps can lead to more pronounced bone remodeling, flapless techniques are less traumatic, do not interrupt the vascularization of the area and also preserve the keratinized mucosa [28].

Similar advantages were reported when using another technique that avoids flap mobilization after extraction with the socket plug technique, using lyophilized inorganic bovine bone, resorption after three months being limited to 12–14% of the initial bone width, while the control group had a resorption of 21% [29].

Our results are in accordance with the previous reported results [30], when the “ice cream cone” technique was evaluated. Other studies have shown that, on average, the reduction in the width of the alveolar ridge was 4.0 mm and went up to 50% of the size measured before extraction. These studies showed rapid reductions in the first six months, followed by gradual decrease in size at subsequent evaluations. Moreover, it was observed that in extractions without bone preservation, the horizontal reduction was greater than the vertical reduction of hard tissue in the first 12 months [31,32]. Consequently, the “ice cream cone” technique ensures a soft tissue manipulation that allows secondary healing, reducing the average value of the buccal-oral resorption of the alveolar ridge to only 1.32 mm [30]. Comparing the results of this technique with those obtained with the “open healing” technique, we observe a reduction of the vestibular-oral width only from T_0_ to T_1_, while from T_1_ to T_5_ there was no statistically significant difference between the measured values (after loading and up five years follow up).

Regarding complications, we found no connection between the number of walls of the defect (Figure 1 and Figure 2) and the occurrence of complications during the healing period. This result completes the conclusions issued by Taffet [9], which show that the higher the number of bone walls that delimit the defect, the fewer complications occur during healing. Our report found no association between the number of walls that delimit the defect before the extraction and the type of intervention for implant insertion. In the sites where we used “open healing” together with the immediate implantation, we noticed that the bone surrounding the implant showed dimensional stability.

Limitation of the study: We acknowledge the lack of a control group might be regarded as a limitation, as could the heterogeneity of the collected data set in terms of surgical site location (upper/lower jaw, anterior/posterior), defect types and tooth morphology (single/multiple root) that were all suitable for the open healing protocol.

Prospective studies with control groups are needed to further investigate this surgical approach. The prospective studies could compare the outcome of open and closed healing under standardized clinical conditions.

## 5. Conclusions

Minimally invasive flapless membrane assisted “open healing” technique is safe and relies on biological principles. Being a flapless technique, it has the advantages of maintaining the blood supply at the bone-periosteum interface, preserving the three-dimensional architecture of both hard and soft tissues surrounding the initial defect. This technique reduces the morbidity related to the flap protocols, generating patients an intra- and post operatory comfort.

CBCT measurements confirmed stabile bone height, both buccal and palatal/lingual, with no significant statistical differences in all time frames up to five years after loading follow up. 

The technique allows implant placement either in the same stage with the flapless atraumatic extraction, when a predictable esthetic and functional outcome is achievable, or with a flapless approach in a later stage.

## Figures and Tables

**Table 1:** Evaluation timepoints.

Timepoints	Description
T_0_	initial situation
T_1_	before loading for the immediate implant sites (**N_1_ group**)
	before implant insertion (**N_2_ group**)
T_2_	loading time
T_3_	12 months after loading
T_4_	24 months after loading
T_5_	60 months after loading

**Table 2:** Distribution of surgical sites. Stage 1: initial surgery (extraction with or without implant placement).

	Stage 1	Extraction + Implant N_1_ Group	Extraction Only N_2_ Group	Total N
Jaw				
Upper jaw		14	71	85
Lower jaw		12	38	50
total		**N_1_ = 26**	**N_2_ = 109**	**N = 135**

**Table 3:** Buccal (vestibular) wall bone height (CBCT measurements, mm)—Summary statistics. SD: standard deviation, IQR: interquartile range.

Moment	N	Mean	SD	Median	Minimum	Maximum	IQR	Normal Distribution (*p*)
T_0_	135	11.895	3.352	11.77	3.70	21.74	9.557–14.010	0.2843
T_1_	105	12.534	3.086	12.15	5.31	22.74	10.403–14.840	0.1080
T_2_	131	12.613	2.837	12.50	5.30	21.70	10.578–14.813	0.3678
T_3_	132	12.517	2.712	12.40	5.30	21.59	10.605–14.450	0.2321
T_4_	128	12.422	2.679	12.27	5.35	21.50	10.500–14.290	0.1791
T_5_	125	12.205	2.713	12.02	4.99	21.45	10.388–14.188	0.1825

**Table 4:** Oral (palatal/lingual) wall bone height (CBCT measurements, mm)—Summary statistics. SD: standard deviation, IQR: interquartile range.

Moment	N	Mean	SD	Median	Minimum	Maximum	IQR	Normal Distribution (*p*)
T_0_	135	13.093	3.399	13.30	3.25	21.58	11.202–15.185	0.4261
T_1_	105	12.935	3.089	12.71	5.79	22.39	10.992–14.788	0.0779
T_2_	131	12.908	2.844	12.91	5.71	21.33	11.000–14.473	0.3428
T_3_	132	12.843	2.835	12.75	5.69	21.27	10.905–14.395	0.1898
T_4_	128	12.694	2.757	12.49	6.00	21.30	10.815–14.215	0.1330
T_5_	125	12.554	2.763	12.44	6.00	21.23	10.550–14.145	0.1244

**Table 5:** Alveolar ridge width (CBCT measurements, mm)—Summary statistics. SD: standard deviation, IQR: interquartile range.

Moment	N	Mean	SD	Median	Minimum	Maximum	IQR	Normal Distribution (*p*)
T_0_	135	8.241	2.052	8.10	3.30	15.07	6.912–9.510	0.1581
T_1_	105	7.653	1.873	7.50	2.58	13.04	6.380–8.820	0.2940
T_2_	131	7.405	1.860	7.01	3.92	14.77	6.000–8.400	0.0781
T_3_	132	7.378	1.849	6.97	4.02	14.80	6.025–8.445	0.0971
T_4_	128	7.336	1.829	6.99	4.14	14.66	6.015–8.385	0.1102
T_5_	125	7.430	1.948	7.00	4.10	14.51	6.057–8.525	0.0853

## Data Availability

The data presented in this study are available on request from the corresponding author.
